# Overview of the First 6 Months of Clinical Trials for COVID-19 Pharmacotherapy: The Most Studied Drugs

**DOI:** 10.3389/fpubh.2020.00497

**Published:** 2020-08-21

**Authors:** Maria Laura Idda, Dorian Soru, Matteo Floris

**Affiliations:** ^1^Institute for Genetic and Biomedical Research, National Research Council, Sassari, Italy; ^2^Independent Consultant, Ovodda, Italy; ^3^Department of Biomedical Sciences, University of Sassari, Sassari, Italy

**Keywords:** clinical trial (2.172), COVID-19, coronavirus (2019-nCoV), COVID-19 (condition), COVID-19 infection

## Abstract

SARS-CoV-2 rapidly spread from China until it was defined a pandemic by WHO in March 2020. Related scientific papers have rapidly extended information regarding the diagnosis, treatment and epidemiology of COVID-19 infection. To date, no vaccine or definitive treatment is available to defeat the virus and therapies are mainly based on existing drugs used to treat other conditions. Existing therapies used in several clinical trials work by affecting the biology of COVID-19 and/or counteracting the harmful host excessive immune response. Here, we have reviewed 526 ongoing clinical trials for COVID-19 to provide a perspective on the first 6 months of global efforts to identify an effective therapy. The drugs most actively tested in various centers include hydroxychloroquine, ritonavir, azithromycin, tocilizumab, lopinavir chloroquine and ivermectin. Our analysis shows that most clinical trials focus on a small number of candidate drugs (namely hydroxychloroquine and chloroquine representing 25% of total clinical trials) while underestimating the potential of other promising drugs. A global coordination in clinical trial management could avoid duplications and increase the effectiveness of the response to the global challenge.

## Introduction

The outbreak of the coronavirus disease 2019 (COVID-19) has generated a global health issue. COVID-19 is a pathogenic viral infection caused by severe acute respiratory syndrome coronavirus 2 (SARS-CoV-2), which appeared in 2019 in Wuhan, China. From a pathological point of view, the most common symptoms observed during COVID-19 infection are fever (83.3%), cough (60.3%), dyspnea, and myalgia or fatigue; and anosmia and ageusia are also commonly observed (1, 2). Furthermore, gastrointestinal symptoms could also be initial manifestations of COVID-19 and contribute to the diffusion of the virus through fecal samples, especially in children (3). More recently, development of venous thromboembolism in patients with COVID-19 has been reported (4).

SARS-CoV-2 is a betacoronavirus, one of the four genera of coronaviruses, belonging to the same sub-group as the Severe Acute Respiratory Syndrome-CoV (SARS-CoV, SARS outbreak in 2002) and the Middle East Respiratory Syndrome-CoV (MERS-CoV, MERS outbreak in 2012) (5). Generally, coronaviruses are extremely small (65–125 nm in diameter) and contain a single-stranded RNA ~26–32 Kbs long (6). All coronavirus genomes are organized as follows: a 5′-untranslated region (5′-UTR), open reading frame (orf) 1a/b encoding proteins necessary for virus replication, downstream genes encoding structural proteins including *spike*, and elements necessary for the envelop, membrane, and nucleocapsid production; finally, accessory proteins and the 3′-untranslated region (3′-UTR) (7). *Spike* is a glycoprotein located on the outer surface of coronaviruses that is responsible for the attachment and entry of the virus to host cells. After binding of *spike* to the human receptor angiotensin-converting enzyme 2 gene (ACE2), a conformational change in the spike protein facilitates the fusion of the viral envelope with the cell membrane through the endosomal pathway (8). Then SARS-CoV-2 releases RNA into the host cell. Genome encoding begin following RNA entering to the host cell and enables the expression of proteins, which progress the adaptation of the virus to the human host. Importantly, the entry mechanism of coronavirus is strongly dependent on cellular proteases. For coronavirus such as the SARS-CoV, the transmembrane protease serine 2 (TMPRSS2) and cathepsin play a critical role in virus entry, they split the *spike* protein and begin all the changes necessary for the virus penetration (9). Recently it was reported that SARS-CoV-2 may use a similar mechanism and that SARS-CoV-2 cell entry may be facilitated by ACE2 and TMPRSS2 (10, 11).

In addition to a growing knowledge of molecular mechanisms, new information regarding diagnosis, treatment and epidemiology of COVID is rapidly accumulating, permitting greater understanding of the disease pathway and progression and identification of new pharmacological targets. While numerous clinical trials are on-going to identify therapeutic approaches by repurposing existing drugs, today the main international response to COVID-19 is mainly limited to contain disease spread. The need to identify innovative treatment strategies remains a priority.

Here, we reviewed 526 ongoing clinical trials (last update: July 6, 2020) to offer a view on the first 6 months of efforts to identify an effective therapy for COVID-19. A large number of drugs (265) are under investigation, but current efforts are biased toward a limited number of them. Indeed, the great majority of clinical trials are focused on a small number of candidate drugs including, hydroxychloroquine, ritonavir, azithromycin, tocilizumab, lopinavir chloroquine and ivermectin (12), while potentially and more promising ones are less considered. For example, host-directed therapies such as those based on inhibitors of the human serine protease TMPRSS2 (bromhexine, camostat, and nafamostat) are considerably less explored. Conversely, there are conflicting and discordant results on hydroxychloroquine, the most tested drug (about 1 of 5 trials). Global coordination of clinical trials could avoid current redundancy and potentiate the effort to explore other possibilities.

## Results and Discussion

The current COVID-19 pandemic boosted the growth of new pharmaceutical research programs and the proliferation of a large number of clinical trials worldwide. Indeed, researchers are attempting to identify drugs to treat the disease using different approaches including repurposing of existing drugs, high throughput screening and virtual screening of new compound; the use of natural and traditional products have also been evaluated. Repurposing of existing drugs, the identification of a new medical use, in this case antiviral activity, for already known drugs, including approved, and discontinued one, is playing a key role in this effort. Initially, interferons nebulization and anti-viral drugs were used to reduce the viral load. Type I interferons (IFNs) inhibit the replication of both DNA and RNA viruses at different stages of their replicative cycles and have strong antiviral activity (13, 14). Unfortunately, only remdesivir, an antiviral drug with nucleotide analog activity has demonstrated relevant antiviral activity. Preliminary observations from a multicentric study, in a cohort of 53 patients hospitalized for severe Covid-19 who were treated with compassionate-use remdesivir, demonstrated clinical improvement in 68% of patients (15). More recently, a larger double-blind, randomized, placebo-controlled trial demonstrated that intravenous remdesivir is superior to placebo in shortening the time to recovery in adults hospitalized with Covid-19. Furthermore, the same study estimated that 14-days mortality was 7.1% with remdesivir and 11.9% with placebo (16).

**Figure 1:**
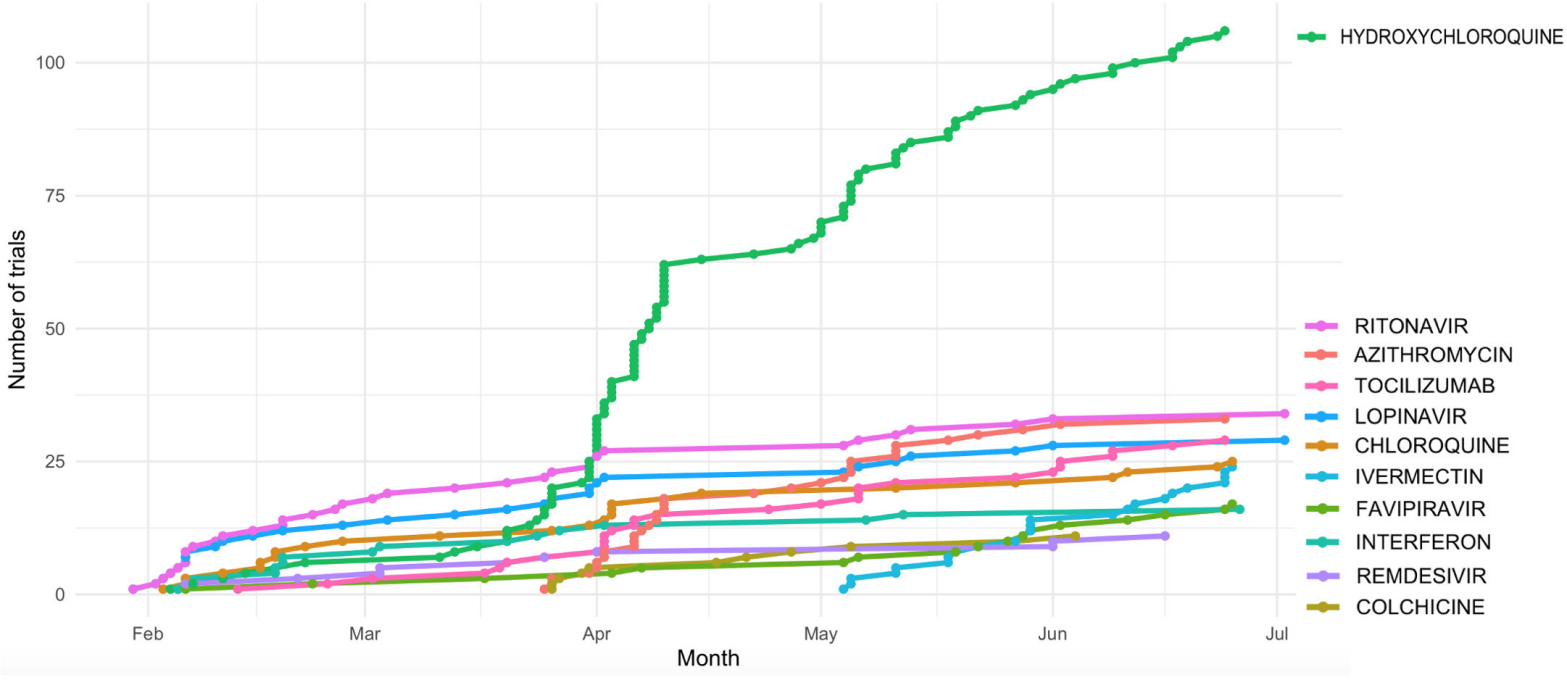
Cumulative number of clinical trials of the most actively tested drugs registered during the first 6 Months after the first COVID-19 published trial (23 Jan 2020).

To have a complete picture of the ongoing trials to treat COVID-19 infection, we collected a comprehensive list of COVID-19 clinical trials from the 2 main public repositories, as of July 6th, 2020 (**Methods**). We then made coherent the names of the drugs (for example, different salts of the same active principle were considered as one single drug) provided by the different sources (**Methods**) and obtained a final list of 526 clinical trials that were analyzed. Most trials focus on a restricted number of drugs, including hydroxychloroquine and antivirals previously used for treatment of other viral infection, mainly HIV (Figure 1 and Table 1). Of note is the use of anti - inflammatory molecules which prevent adverse effects related to over-reactive immune system.

**Table 1:** Description of the drugs tested in at least 10 COVID-19 clinical trials as of July 8, 2020.

**Drug**	**Number of trials**	**Description**	**References**
HYDROXYCHLOROQUINE	106	Treatment of uncomplicated malaria, rheumatoid arthritis, chronic discoid lupus erythematosus, and systemic lupus erythematosus. Hydroxychloroquine accumulation in human organelles also raise their pH, which inhibits antigen processing, prevents the alpha and beta chains of the major histocompatibility complex (MHC) class II from dimerizing, inhibits antigen presentation of the cell, and reduces the inflammatory response. The raised pH in endosomes, prevent virus particles (such as SARS-CoV and SARS-CoV-2) from utilizing their activity for fusion and entry into the cell	(17)
RITONAVIR	33	HIV protease inhibitor that interferes with the reproductive cycle of HIV; more commonly used as a booster of other protease inhibitors. For example, Ritonavir is a potent inhibitor of the enzymes responsible for lopinavir metabolism, and its co-administration “boosts” lopinavir exposure and improves antiviral activity	(18)
AZITHROMYCIN	33	Antibiotic used for the treatment of a number of bacterial infections	
TOCILIZUMAB	29	Recombinant, humanized, anti-human interleukin 6 (IL-6) receptor monoclonal antibody	(19)
LOPINAVIR	29	Antiretroviral protease inhibitor used in combination with other antiretrovirals in the treatment of HIV-1 infection	(20)
CHLOROQUINE	25	See HYDROXYCHLOROQUINE	
IVERMECTIN	24	This drug has a broad-spectrum activity with high lipid solubility and possesses numerous effects on parasites, nematodes, arthropods, flavivirus, mycobacteria, and mammals through a variety of mechanisms	(21)
FAVIPIRAVIR	17	A pyrazine analog initially approved for therapeutic use in resistant cases of influenza. The antiviral targets RNA-dependent RNA polymerase (RdRp) enzymes, which are necessary for the transcription and replication of viral genomes	(22)
INTERFERON	16	First cytokines produced during a viral infection; inflammation, signaling and immunomodulation	(23)
REMDESIVIR	11	Remdesivir is a nucleoside analog that is expected to inhibit the action of RNA polymerase	
COLCHICINE	11	Inhibits the hepatitis C NS5B protein, RNA-dependent RNA polymerase	(24)

Hydroxycloroquine (*N* = 106 clinical trials), Azithromycin (*N* = 33), the antiviral compounds Ritonavir and Lopinavir (*N* = 33 and 29 clinical trials, respectively), and Tocilizumab (*N* = 29) are among the drugs more actively tested. Ritonavir and Lopinavir – a classical HIV first-line therapy - are usually administered in combination. They are followed by Chloroquine (*N* = 25 trials) and Ivermectin (*N* = 24). The distribution of the number of clinical trials per drug is significantly skewed toward such low number of drugs (*p* < 0.001, 1 DF Chi square test).

Chloroquine and its derivative Hydroxychloroquine are widely used to treat malarial infection and selected inflammatory conditions such as autoimmune disease (25). Multiple lines of evidence have suggested that chloroquine has the capacity to inhibit the replication of several micro-organisms, including coronaviruses such as SARS-CoV-2, *in vitro* (17). Today, hydroxychloroquine and chloroquine are under investigation in clinical trials for both, prophylaxis in pre-exposure to virus and treatment post-exposure to SARS-CoV-2 (26). Many hospitals are currently using hydroxychloroquine as first-line therapy for hospitalized patients with COVID-19, and on March 29 FDA issued authorization for 30 million doses of hydroxychloroquine and chloroquine donated by Sandoz. Unfortunately, clinical data supporting the effectiveness of these two drugs are still inconclusive. The efficacy of hydroxychloroquine was supported by a small trial with 62 patients suffering from severe COVID-19 diagnosed and admitted to Renmin Hospital of Wuhan University (27). Later, a smaller pilot study at the Shanghai Public Health Clinical Center (28) demonstrated its activity against SARS-CoV-2, although its use was subsequently discouraged by a smaller study with just 11 patients from a clinical study performed in a French hospital (29). Beyond the lack of data on the real effectiveness of these drugs until the middle of July, the possibility of side effects as a result of their use is well-known, especially when provided in combination with other drugs. A group of cardiologists in New York, for example, found notable signs of QT interval prolongation in 30% in a group of 84 COVID-19 patients treated with hydroxychloroquine and azithromycin (30).

The main antiretroviral drugs studied in the world against COVID-19 are Ritonavir and Lopinavir, two antivirals often used in combination as first-line therapy against HIV. Interestingly, the largest study in hospitalized adult patients with severe Covid-19 has shown no benefit as compared with standard care after lopinavir –ritonavir treatment (20). Although, even in this case the data supporting the efficacy are unfavorable, regulatory agencies have approved the use of this combination therapy, limiting it to less severe COVID-19 patients ^1^.

Other antiviral compounds among the most tested drugs are Favipiravir, Umifenovir and Oseltamivir. Favipiravir has been approved in Japan and China for the treatment of novel influenza virus infections; its efficacy has been only weakly documented by a paper later retracted (31) and by a preprint article (32). Umifenovir (trade name: Arbidol) is a dual-acting direct antiviral/host-targeting agent (33); it is under evaluation in 12 clinical trials, and to date only 2 small-scale studies tested its efficacy in comparison with a Lopinavir/Ritonavir based treatment (34, 35). Finally, during a clinical trial to test the effectiveness of Oseltamivir the authors noted no favorable outcomes against SARS-CoV-2 (36).

As mentioned above, the most promising antiviral compound tested for COVID-19 is Remdesivir.

The immunosuppressant anti-IL6 Tocilizumab (37), used for the treatment of rheumatoid arthritis, is the most widely tested drug directed against a human target. Several reports have identified elevation of IL-6 levels in critically ill COVID-19 patients, as compared with that of survivors and those with less severe disease (1). Consistent with this finding and with the efficacy to restrict the IL-6 pathway, Tocilizumab is tested in 29 trials. Tocilizumab is approved for the treatment of severe or life-threatening cytokine-release syndrome caused by chimeric antigen receptor T-cell therapy (37). Additionally, tocilizumab also has FDA-approved indications for giant cell arteritis, polyarticular juvenile idiopathic arthritis, and systematic juvenile idiopathic arthritis. Until now, tocilizumab was not officially approved by the FDA for use in COVID-19 treatment and few published data pertain to the safety or efficacy of this drug for COVID therapy. Other immunosuppressive agents, Anakinra (anti-IL1) and Sarilumab (anti-IL6 receptor) are being tested in 8 and 5 different trials, respectively.

Surprisingly, none of the drugs directed against the mechanism of viral entry into human cells are among the most tested drugs. In particular, we observed only 5 and 4 trials for the serine protease inhibitors Camostat and Bromhexine, respectively, and 1 single trial for the analog Nafamostat (Supplemental Table S1).

We also identified several clinical trials where drug-drug interaction alerts should be considered when the combinations are proposed in the same trial (Table 2). Among others, Hydroxychloroquine (HCQ) is frequently tested with Lopinavir (which increases the serum levels of HCQ) and with Ritonavir (whose serum levels are increased by the concomitant administration of HCQ).

**Table 2:** Drug combinations tested in clinical trials where drug-drug interaction alerts are reported in the Drugbank “Drug*-Drug Interaction Checker*.”

**Drug A**	**Effects**	**Drug B**
Azithromycin	Increases risk or severity of QTc prolongation of	Hydroxychloroquine
Daclatasvir	Increases serum levels of	Sofosbuvir
Favipiravir	Lowers metabolism rate of	Chloroquine
Hydroxychloroquine	Increases serum levels of	Ritonavir
Lopinavir	Lowers excretion rate & increases serum levels of	Emtricitabine
Lopinavir	Increases serum levels of	Hydroxychloroquine
Ritonavir	Lowers excretion rate & increases serum levels of	Sofosbuvir

Based on the current data, it is evident that mainly repurposed antiviral drugs (whose function is not yet guaranteed) are tested for COVID-19 treatment. Interestingly, drugs directed against the virus entry and replication mechanism (including in particular host-directed-therapies such as *nafamostat mesylate* and the analogous *camostat mesylate*, or the *recombinant ACE2 protein*) are tested less frequently, although they have a mechanism of action intimately and directly involved in the biology of the infection.

The use-abuse of repurposing could be one of the main reason for COVI-19 trials failure. What is sure, especially in clinical trials, is that good results must be obtained with slow and careful experiments to be reliable and secure for population. Remdesivir for example, initially developed against hepatitis C, which showed great potential against zoonotic viruses including SARS and MERS, have been found to help COVID-19 patients to recover faster. The drug did not work against hepatitis C as expected but researchers established that Remdesivir is safe for humans. Thus, after COVID-19 outbreak, researchers could quickly roll out clinical trials to test Remdesivir for Covid-19. This example clearly shows that the reason why most clinical trials are looking to repurpose existing drugs is mainly related to the possibility to faster use them for human patients escaping months or years of safety testing.

Another reason of these failures could be the existence of a perverse mechanism, where the choice of priorities in drug testing is led by small uncontrolled studies that fuelled a strong pressure by media, politicians and not by strong scientific evidences strongly contribute to this problem. Indeed, 6 months after the first clinical trial the number of coronavirus cases are still rising and nothing seems to be able to work as an effective Covid-19 treatment.

This obvious bias, generating overlapping studies, could only be overcome by global coordination of clinical trial policies, which could also help to avoid redundancy that also slows the identification of effective therapies.

## Methods

### Data Collection

We collected a comprehensive list of COVID-19 clinical trials from 2 different public repositories (ClinicalTrials.gov and WHO ICTRP - International Clinical Trials Registry Platform, both accessed on July the 6th, 2020), using the search keywords “COVID-19, SARS-CoV-2, severe acute respiratory syndrome coronavirus, 2019-nCoV, 2019 novel coronavirus, Wuhan coronavirus,” and considering only pharmacological interventions where one or more drugs are explicitly listed. From ClinicalTrials.gov (https://clinicaltrials.gov/ct2/results?cond=COVID-19) we retrieved 2.427 studies, of which 1.358 were interventional studies. From WHO ICTRP (International Clinical Trials Registry Platform, https://www.who.int/ictrp/en/) we retrieved 654 studies, of which 335 were interventional studies.

Based on the identifiers assigned to each trial, duplicate entries were considered only once.

**Figure 2:**
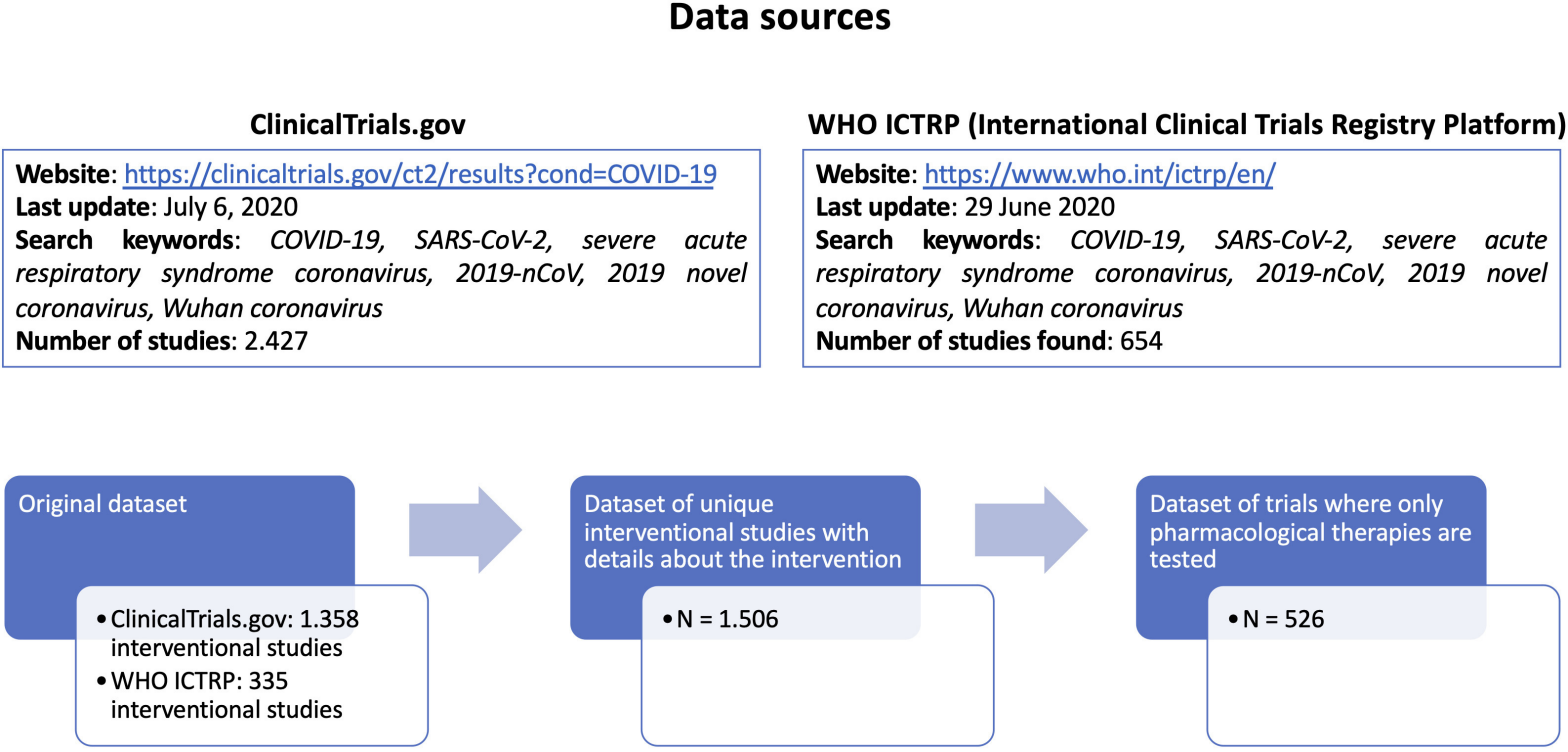
Flow chart with the detailed description of data retrieval and processment.

The merged dataset contained 1.693 unique interventional studies, of which, 1.506 have complete intervention details (Figure 2).

### Analysis

From the raw data we kept only trials where the following informations were clearly available: trial recruitment status, list of participating countries, name of the drug(s), clinical trial phase. We then performed a standardization of the information provided by the different sources. Furthermore, for each active ingredient we retrieved the corresponding DrugBank identifier (38) to retrieve drug-related informations.

We excluded from our analysis all the therapies whose active ingredients were not clearly declared, and therapies based on nutraceuticals and traditional medications.

We obtained a final list of 526 clinical trials considered for the analysis (Supplemental Table S1).

The non-random nature of the distribution of number of trials per drug has been checked with a 1DF chi square test using the R package for statistical analysis, that returned a *p* < 0.001.

Considering that many drugs are tested in combinations, we checked whether the concomitant administration of these drug could be problematic using the *Drug-Drug Interaction Checker* [https://www.drugbank.ca/interax/multi_search], a freely available resource reporting data from clinical guidelines, labels and scientific literature, and covering approved drugs by the Food and Drug Administration (FDA), Health Canada and the European Medical Association (EMA).

## Data Availability Statement

All datasets generated for this study are included in the article/Supplementary Material.

## Author Contributions

MI: conceptualization and writing–original draft. DS: data curation. MF: conceptualization, writing–original draft, and supervision. All authors contributed to the article and approved the submitted version.

## Conflict of Interest

The authors declare that the research was conducted in the absence of any commercial or financial relationships that could be construed as a potential conflict of interest.

## Footnotes

^1^Available online at: http://www.salute.gov.it/portale/news/p3_2_1_1_1.jsp?lingua=italiano&menu=notizie&p=dalministero&id=4395.
